# The Synergistic Effect of Combined Immunization with a DNA Vaccine and Chimeric Yellow Fever/Dengue Virus Leads to Strong Protection against Dengue

**DOI:** 10.1371/journal.pone.0058357

**Published:** 2013-03-05

**Authors:** Adriana S. Azevedo, Antônio J. S. Gonçalves, Marcia Archer, Marcos S. Freire, Ricardo Galler, Ada M. B. Alves

**Affiliations:** 1 Laboratório de Biotecnologia e Fisiologia de Infecções Virais, Instituto Oswaldo Cruz, Fundação Oswaldo Cruz, Rio de Janeiro, Brazil; 2 Instituto de Tecnologia em Imunobiológicos, Fundação Oswaldo Cruz, Rio de Janeiro, Brazil; University of Delhi, India

## Abstract

The dengue envelope glycoprotein (E) is the major component of virion surface and its ectodomain is composed of domains I, II and III. This protein is the main target for the development of a dengue vaccine with induction of neutralizing antibodies. In the present work, we tested two different vaccination strategies, with combined immunizations in a prime/booster regimen or simultaneous inoculation with a DNA vaccine (pE1D2) and a chimeric yellow fever/dengue 2 virus (YF17D-D2). The pE1D2 DNA vaccine encodes the ectodomain of the envelope DENV2 protein fused to t-PA signal peptide, while the YF17D-D2 was constructed by replacing the prM and E genes from the 17D yellow fever vaccine virus by those from DENV2. Balb/c mice were inoculated with these two vaccines by different prime/booster or simultaneous immunization protocols and most of them induced a synergistic effect on the elicited immune response, mainly in neutralizing antibody production. Furthermore, combined immunization remarkably increased protection against a lethal dose of DENV2, when compared to each vaccine administered alone. Results also revealed that immunization with the DNA vaccine, regardless of the combination with the chimeric virus, induced a robust cell immune response, with production of IFN-γ by CD8+ T lymphocytes.

## Introduction

Dengue is an important viral disease, consisting of a global public health problem in tropical and subtropical regions of the world including the Americas, where the main vector is the mosquito *Aedes aegypti*. It is estimated that over 2.5 billion people live in areas of dengue risk in which 50 to 100 million of infection occur annually and about 250 to 500 thousand patients develop the most severe symptoms of the disease, such as dengue hemorrhagic fever (DHF) and dengue shock syndrome (DSS), with more than 20,000 deaths [1], [2]. Despite several efforts, there is still neither an effective antiviral therapy nor a preventive vaccine against dengue commercially available [3].

There are four antigenically distinct dengue viruses (DENV1-4), which belong to the family Flaviviridae, genus Flavivirus [4]. The DENV genome is a positive single-stranded RNA, encoding a polyprotein which is processed to produce three structural proteins, capsid (C), premembrane/membrane (prM/M) and envelope (E) and seven nonstructural (NS) proteins, NS1, NS2A, NS2B, NS3, NS4A, NS4B and NS5 [5]. The E glycoprotein is the major component of virion surface and it is associated with several biological activities. It acts as a binding protein, interacting with receptors present on host cell surface and leading to endocytosis of the virus particle. It also mediates fusion of envelope and host cell membranes, which culminates with the nucleocapsid disassemble and release of virus genome for polyprotein synthesis [5], [6]. The virus particle contains 90 homodimers of the E protein and its ectodomain is composed of the domain I, II and III [6].

This protein is the main target for the induction of neutralizing antibodies and therefore most vaccines being developed against DENV are based on the stimulation of immune responses towards the E glycoprotein [7], [8]. One of the main problems for developing a vaccine against dengue is the requirement for a protective immune response against all four serotypes, without the risk of inducing severe disease [9], [10]. This rational is particularly attributed to epidemiological observations that most severe dengue cases occur in individuals experiencing a secondary DENV infection [11] and an inefficient immunization against one serotype may increase the risk of DHF/DSS development if the vaccinated host acquires an infection with such serotype.

Several vaccine approaches have been proposed to combat dengue disease, including the use of inactivated or live attenuated viruses, chimeric live viruses, subunit antigens and DNA immunizations [9], [12], [13]. Immunization with tetravalent formulations containing sets of live attenuated viruses lead to unbalanced immune responses against the four serotypes, due to the interference of one virus in the replication of the others and/or the immunodominance of the response against some antigens upon others [9]. In fact, clinical studies with chimeric attenuated viruses by Sanofi-Pasteur, which is the most advanced tetravalent live attenuated dengue vaccine, revealed the necessity of three doses with several month intervals to reach seroconversion against all the four serotypes [14], [15]. On the other hand, the DNA vaccine is a gene-based strategy which seems not to cause interference upon combined immunization [16]. Actually, DNA vaccines have been shown to be significantly more effective when combined in other immunization approaches, such as in prime/boost regimen, leading to a synergistic effect of the immune response that can reduce the number of doses required for protection [17]–[19].

Therefore, in the present work we evaluated the combination of these two vaccine strategies for eliciting a robust immune response and protection against dengue. Balb/c mice were immunized with a DNA vaccine (pE1D2), which encodes the ectodomain of the envelope DENV2 protein, previously constructed by our group [20], combined to a chimeric yellow fever/dengue virus (YF17D-D2) [21]. Mice were inoculated with these vaccines by prime-booster or simultaneous immunization protocols and we observed a synergistic effect on the elicited immune response, increasing neutralizing antibody titers and conferring protection against a lethal challenge with DENV2. Results also revealed that immunization with the DNA vaccine, regardless of the combination with the chimeric virus, induced a robust cell immune response with the production of INF-γ by CD8+ T lymphocytes.

## Materials and Methods

### Viruses and Cell Lines

The DENV2, strain New Guinea C (NGC) (GenBank M29095), was used for construction of the pE1D2 recombinant plasmid. This strain was neuroadapted by passaging in newborn mouse brains and used in challenge assays. The DENV2 44/2 [21] was used for plaque reduction neutralization test. Both viruses were propagated in Vero cell monolayers (ATCC), cultivated in Medium 199 with Earle salts (E199, Sigma, USA), buffered with sodium bicarbonate, supplemented with 5% fetal bovine serum (FBS, Invitrogen, USA) and maintained at 37°C in 5% CO_2_.

### Vaccines

#### The DNA vaccine PE1D2

The DNA vaccine pE1D2 was previously described [20]. Briefly, this plasmid encodes the NGC DENV2 ectodomain (domains I, II and III) of E protein fused to a signal sequence derived from the human tissue plasminogen activator (t-PA). The pcTPA plasmid [22], without the E gene, was used as a control. Plasmids were isolated from transformed *Escherichia coli*, DH5-α strain, purified by Qiagen Endofree Plasmid Giga Kit (Qiagen, Germany) following manufacturer's instruction, suspended in endotoxin-free sterile water and stored at −20°C until use.

#### Chimeric virus YF17D-D2

The YF17D-D2 (P44/S) vaccine is a chimeric yellow fever (YF)/dengue 2 virus, constructed by replacing prM and E genes of the live attenuated virus vaccine YF17D by these genes from DENV2, as described previously [21]. The chimeric virus stock was amplified and tittered on Vero cells and stored at −70°C until use.

### Mice Immunization

Experiments with mice were conducted in compliance with ethical principles in animal experimentation stated in the Brazilian College of Animal Experimentation and approved by the Institute's Animal Use Ethical Committee (approval ID: L-067/08). Groups of Balb/c mice (n = 10), male SPF with 4 to 6 weeks old, were inoculated by three different immunization protocols and challenged with DENV2, as summarized on table 1. The single type protocol was performed by immunization with the DNA vaccine or chimeric virus alone. Briefly, each animal was inoculated with the pE1D2 vaccine or the control plasmid (pcTPA) (100 µg DNA/dose/animal) intramuscularly (i.m.), as described [20], or subcutaneously (s.c.) with the chimeric YF17D-D2 virus (10^5^ PFU/dose/animal). In both cases animals were immunized with one or two doses, given 2 weeks apart. In the prime-booster protocol (DNA/chimera), animals were inoculated, as described above, with one or two doses of plasmid DNA (prime) followed by a booster dose of YF17D-D2, given 10 days after the last DNA dose. Another group was immunized by an inverse prime-booster scheme (chimera/DNA), following the same doses and inoculation routes. Finally, in the simultaneous protocol, mice were inoculated with one or two doses of a mixture with the plasmid DNA and chimeric virus by the intramuscular route (Mix: DNA+chimera), or with two doses of each vaccine by different routes (DR: i.m. and s.c. for plasmid DNA and chimeric virus, respectively), given two weeks apart. Non immunized mice were also used as negative control. Animals were bled by retro-orbital puncture before inoculation (preimmune sera) and one day before challenge. Serum samples were collected and stored at −70°C until use.

**Table 1:** The different immunization protocols using the pE1D2 DNA vaccine and/or YF17D-D2 chimeric virus.

	Vaccines			Challenge^d^	
Groups	0	15	25	30	35
Plasmid 1d (i.m)^a^	pE1D2	–	–	DENV2	–
Plasmid 2d (i.m.)^a^	pE1D2 or pcTPA	pE1D2 or pcTPA	–	DENV2	–
Chimera 1d (s.c.)^a^	–	–	YF17D-D2	–	DENV2
Chimera (s.c.)^a^	YF17D-D2	YF17D-D2	–	DENV2	–
Plasmid 1d (i.m.)+Chimera (s.c.)^b^	pE1D2	YF17D-D2	–	DENV2	–
Plasmid 2d (i.m.)+Chimera 1d (s.c.)^b^	pE1D2 or pcTPA	pE1D2 or pcTPA	YF17D-D2	–	DENV2
Chimera 1d (s.c.)+Plasmid 2d (i.m.)^b^	YF17D-D2	pE1D2	pE1D2	–	DENV2
Mix 1d (i.m.): Plasmid+Chimera^c^	pE1D2 or pcTPA+YF17D-D2	–	–	DENV2	–
Mix 2d (i.m.): Plasmid+Chimera^c^	pE1D2 or pcTPA+YF17D-D2	pE1D2 or pcTPA +YF17D-D2	–	DENV2	–
DR: Plasmid (i.m.)+Chimera(s.c.)^c^	pE1D2 or pcTPA+YF17D-D2	pE1D2 or pcTPA+YF17D-D2	–	DENV2	–

Plasmids: pE1D2 (DNA vaccine) or pcTPA (control).

Chimera: YF17D-D2 (Chimeric virus constructed by replacing the prM and E genes from attenuated yellow fever 17D virus by those from DENV2).

a Single type immunization;

b Combined immunization with prime-booster regimen;

c Combined immunization with simultaneous inoculations;

d Challenge with DENV2 by the intracerebral route.

i.m.: intramuscular route; s.c.: subcutaneous route; Mix: plasmid DNA (pE1D2 or pcTPA)+chimeric virus (YF17D-D2); DR: different routes of inoculations for plasmids (i.m.) and chimeric virus (s.c.).

### Plaque Reduction Neutralization Test (PRNT50)

Neutralizing antibodies against DENV2 were measured using plaque reduction neutralization tests (PRNT) performed in Vero cells, with 96-well plates, as previously described [20]. Briefly, serum samples were serially diluted (from 1∶5 to 1∶640) in 50 µL of E199 medium followed by the addition of approximately 30 PFU of DENV2, and incubated at 37°C for 1 h. Vero cell suspensions were added and plates were incubated at 37°C for 3 h. Cells were overlaid with 100 µL of E199 medium with 3% carboxymethylcellulose and plates were incubated for 7 days at 37°C in 5% CO_2_. Cells were then fixed with 10% formalin, stained with crystal violet and plaques were manually counted. Neutralizing antibody titers were expressed by 50% of plaque reduction (PRNT_50%_). This assay is well established in the laboratory, with reproducible results, and it is used for testing mouse individual serum samples, whose volumes are small.

### Interferon Gamma ELISPOT Assay

Spleen cells from groups of immunized mice (n = 5), isolated 10 days after the last immunization, were used in IFN-γ ELISPOT test. The assay was performed with a 9-mer peptide (SPCKIPFEI), corresponding to 331–339 amino acid residues of the DENV2 E protein, which is an immunodominant CD8+ T cell epitope in Balb/c mice specific for the serotype 2 [23]. Animals were immunized with the DNA vaccine and/or chimeric virus, as summarized in table 1. The IFN-γ ELISPOT mouse set (BD Biosciences) was used as previously described [24], in accordance with the manufacturer’s instruction. Briefly, 96-well plates were coated overnight at 4°C with 5 µg/mL IFN-γ capture monoclonal antibody (100 µL in PBS/well), followed by washing and blocking with supplemented RPMI-1640 medium (Sigma), for 2 hours at room temperature. Splenocytes (2×10^5^ cells/well) were added in 100 µL RPMI-1640 with the subsequent addition of 100 µL of the E peptide (final concentration of 10 µg/mL). Non-stimulated and concanavalin A (Con A, 5 µg/mL) stimulated cells were used as negative and positive controls, respectively. Splenocytes were cultured for 20 h at 37°C in 5% CO_2_. Plates were washed once with water and then with 0.05% Tween 20-PBS (PBST), followed by incubation for 2 h at room temperature with 2 µg/mL of biotinylated IFN-γ detection antibody, diluted in 100 µL PBS with 10% FBS. Plates were washed again with PBST and incubated for 1 h at room temperature with streptavidinhorseradish peroxidase diluted 1∶100. Spots were revealed with AEC substrate reagent set (BD Bioscience) at room temperature and counted with an Immunospot reader (Cellular Technology Ltd, Cleveland,OH) using the Immunospot Software Version 3. Results were expressed as the average from triplicate wells of spot-forming cells (SFC) per 2×10^5^ cells, after subtraction of background values detected in splenocytes incubated only with medium.

#### Lethal challenge with DENV2

Mice were challenged with a mouse brain adapted NGC DENV2, ten or fifteen days after the last immunization. Animals were anesthetized with a mixture of ketamine-xylazine [25] and intracerebrally (i.c.) inoculated with 30 µL of 4.32 log_10_ PFU of DENV2, which corresponds to approximately 4 LD50, diluted in E199 medium supplemented with 5% FBS. Animals were monitored for 21 days for mortality and morbidity. The evaluation of different morbidity degrees was performed using an arbitrary scale ranging from 0 to 3 in each animal group (0 = none, 1 = mild paralyses in one hind leg or alteration of the spinal column with a small hump, 2 = severe paralyses in one hind leg and alteration of the spinal column with a small hump or severe paralyses in both hind legs, 3 = two severe hind leg paralyses and deformed spinal column or death).

#### Statistical analysis

Statistical analyses were performed using GraphPad Prism software (La Jolla, USA), version 5.02. For the analysis of survival and morbidity rates, statistical significances were evaluated by chi-square test, while differences in the degree of morbidity, PRNT_50_ titers and ELISPOT assay were analyzed by Mann-Whitney test. Values were considered significant at P<0.05.

## Results

### Induction of Neutralizing Antibodies against DENV2

**Figure 1:**
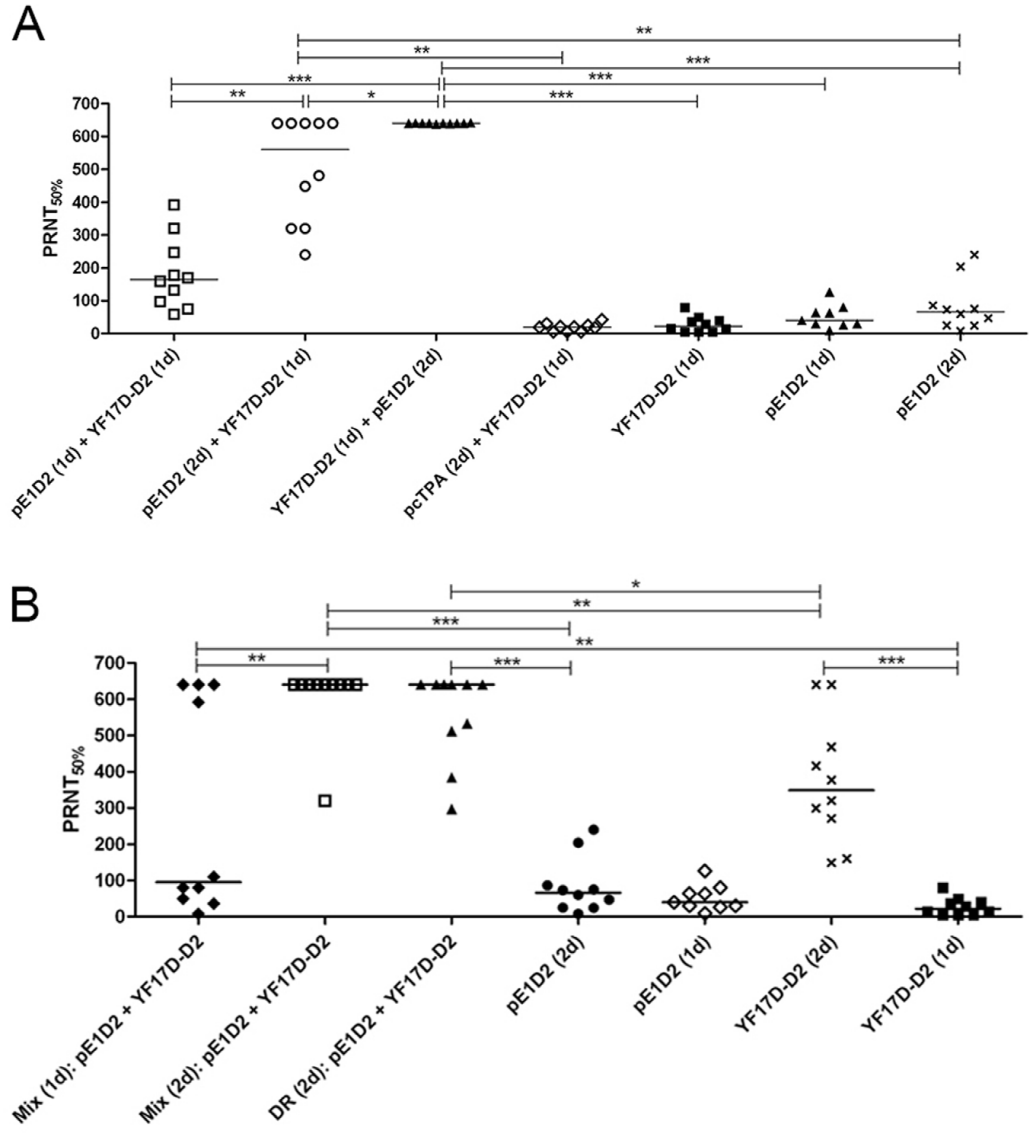
Neutralizing antibody responses against DENV2. Panels A and B refer to samples tested from animals immunized with prime-boost protocols or with simultaneous inoculations, respectively, and control groups. Individual serum samples were collected 24 h before virus challenge. Sera were serially diluted from 1∶5 to 1∶640 and the PRNT_50%_ was performed in 96-well plates. Asterisks indicate differences that are statistically significant using the Mann-Whitney test (*p<0.05; **p<0.01 and ***p<0.001). One dose = 1d; two doses = 2d.

The humoral immune response induced by the DNA vaccine (pE1D2) and/or the chimeric YF/DENV virus (YF17D-D2) was evaluated in Balb/c mice by the presence of neutralizing antibodies against DENV2. Serum samples from mice inoculated with the different immunization protocols (Table 1), as well as control animals, were collected 24 h before virus challenge and titrated for neutralizing antibodies (PRNT_50%_), performed with a DENV2 isolate different from that used for vaccine constructions. Animals inoculated with the single type immunization protocols, only with the pE1D2 DNA vaccine or the YF17D-D2 chimeric virus, showed detectable neutralizing antibodies. Immunization with two doses of the DNA vaccine elicits a slight increase of neutralizing antibody levels when compared to inoculation with one dose (PRNT median of 67 and 40, respectively), while two doses of the chimeric virus induced significant higher PRNT comparing to what it was observed with one dose of this vaccine (median of 349 and 22, respectively) (Fig. 1). However, combined immunizations with the two vaccine strategies revealed an increase in the antibody response. Mice immunized with two doses of the pE1D2 DNA vaccine and boosted with one dose of YF17D-D2 virus presented high antibody titers (median = 560), in which 50% of these animals reached the maximal neutralization titer detected in our assay (≥640) (Fig. 1A). Furthermore, all mice inoculated with the reversed prime-booster protocol, with one dose of YF17D-D2 and two doses of pE1D2, presented the highest neutralizing antibody titer (≥640). On the other hand, animals inoculated with the prime-booster system using the control pcTPA plasmid [pcTPA (2d)+YF17D-D2 (1d)] did not reveal any increase in the humoral immune response (median = 20). In this group, the observed neutralizing antibody titers were, in fact, due to the immunization with the chimeric virus, as can be seen when we compare PRNT in mice receiving only one dose of the YF17D-D2 (median = 22) (Fig. 1A). The immunization scheme with one dose of each vaccine, pE1D2 (prime) and YF17D-D2 (booster), induced significantly lower neutralizing antibody titers (median = 165) when compared to groups that received the former prime-boost protocol, with two doses of pE1D2 and one dose of YF17D-D2 (Fig. 1A). In addition, the simultaneous immunization protocols (Mix), with both vaccines combined in a single formulation, also elicited high levels of neutralizing antibodies, either with one or two doses. In fact, 40% and 90% of animals inoculated with one or two doses of the Mix, respectively, showed the highest antibody titers detected in our assay (≥640) (Fig. 1B). Thus, the scheme of two doses of mixed pE1D2 DNA vaccine and YF17D-D2 virus revealed to be as effective in generating neutralizing antibody levels as one dose of live YF17D-D2 virus followed by two booster doses of the DNA vaccine. In addition, simultaneous immunization with the two vaccines inoculated by different routes (DR) also elicited high neutralizing antibody levels, in which 60% of animals presented maximum PRNT (titers ≥640) (Fig. 1B). Pre-immune serum samples and sera collected from pcTPA-inoculated mice [pcTPA (2d)] did not present detectable neutralizing antibody titers against DENV2 (data not shown).

### Production of IFN-γ Elicited by the Different Immunization Protocols

**Figure 2:**
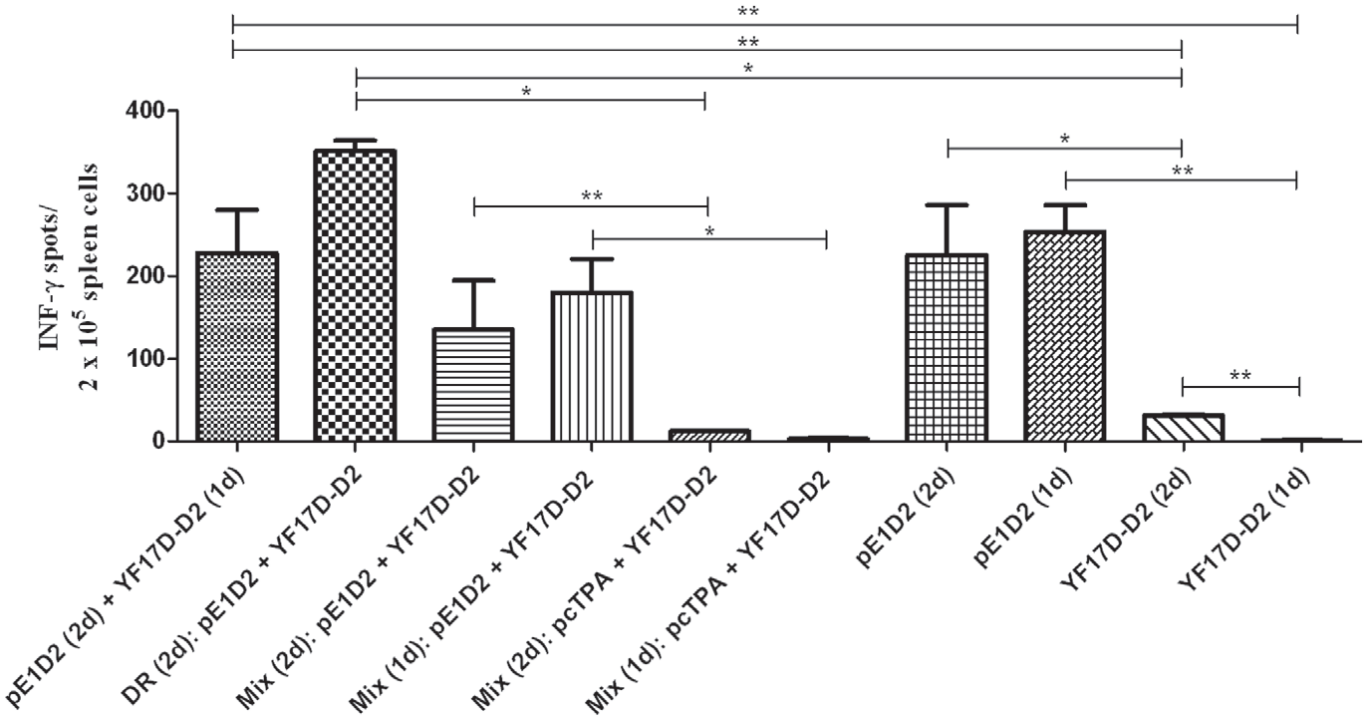
Production of IFN-γ by CD8+ T cells from mice immunized with different vaccination protocols. Splenocytes collected from the different animal groups (n = 5) were stimulated with a T-cell specific peptide and the number of spot-forming cells (SFC) were quantified in a 24 h ELISPOT assay. Asterisks indicate statistically significant differences using the Mann-Whitney test (*p<0.05; **p<0.01). One dose = 1d; two doses = 2d.

The IFN-γ ELISPOT assay was performed in order to investigate CD8+ T cell responses induced by the DNA vaccine and/or chimeric virus, with different immunization protocols (Table 1), using the DENV2 specific E_331–339_ immunodominant epitope, previously described in Balb/c mice [23]. Splenocytes from animals immunized with two doses of the live YF17D-D2 chimeric virus presented significantly higher SFC values when compared to cells collected from mice inoculated with only one dose of such vaccine (Fig. 2). However, mice administered with the pE1D2 DNA vaccine alone, regardless the number of dose (one or two doses), presented a remarkable increase of this immune response, with approximately seven fold more IFN-γ positive SFC numbers when compared to results observed with YF17D-D2-inoculated animal groups (Fig. 2). On the other hand, combined immunizations with these two vaccines, either with the prime-boost scheme or with simultaneous inoculation in a single formulation (Mix) or with different routes (DR), also elicited high numbers of SFC secreting IFN-γ (Fig. 2). Positive control, using Con A as stimulating antigen, confirmed the cell viability from all mouse groups (data not shown).

### Protection against DENV2 Challenge

The protective efficacy of the different immunization protocols were evaluated in vaccinated animals challenged with a lethal dose of a mouse brain-adapted DENV2. Mice were monitored the following 21 days after challenge for the development of morbidity and mortality. The different morbidity degrees were evaluated using an arbitrary scale, regarding mainly hind leg paralysis, alterations in spinal column and death.

**Figure 3:**
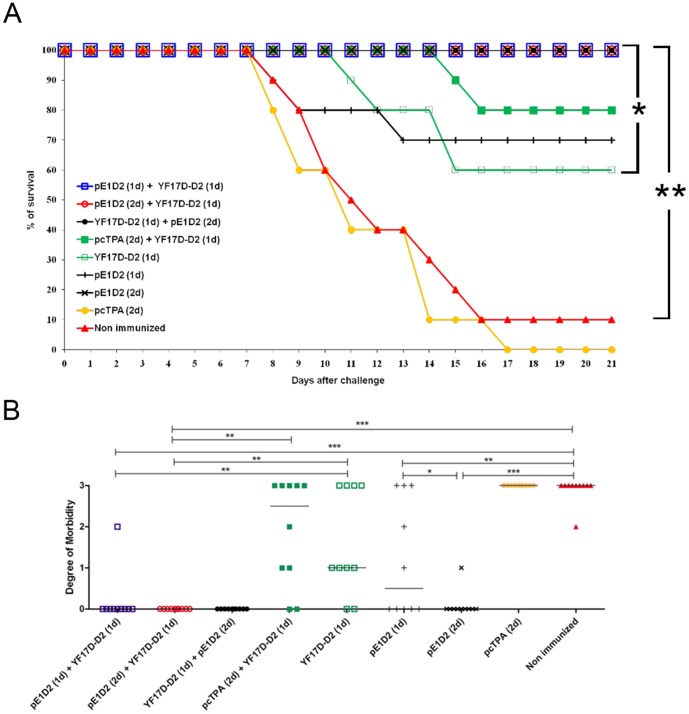
The protective efficacy of vaccination with pE1D2 and YF17D-D2 against DENV2 in prime-booster regimen. Groups of Balb/c mice (n = 10) were immunized with pE1D2 (i.m.) and/or YF17D-D2 (s.c.), administered singly or in a prime-booster regimen, and challenged (i.c.) with the NGC DENV2. Vaccinated and control mice were monitored for 21 days after challenge with the record of survival rates (A) and degree of morbidity (B). Asterisks indicate statistically significant differences using the chi-square test in (A) (*p<0.001; **p<0.0001) and the Mann-Whitney test in (B) (*p<0.05; **p<0.01, ***p<0.001). One dose = 1d; two doses = 2d.

Results indicated that all animals immunized with a prime-booster protocol, with one or two doses of the pE1D2 (prime) followed by a booster dose with YF17D-D2 or in the reverse scheme (one dose of YF17D-D2 and two doses of pE1D2), survived virus challenge (Fig. 3A). Similar results were also observed in mice immunized with two doses of pE1D2 vaccine and all these groups showed statistically significant differences of survival rates (p<0.0001) when compared to control groups (non-immunized and pcTPA-inoculated animals).

However, only animals vaccinated with the prime-booster protocol, with either two doses of pE1D2 and one dose of YF17D-D2 or with the reverse scheme, revealed a total absence of morbidity after virus challenge (Fig. 3B). Additionally, only one mouse (1/10) in the group immunized with one dose of each vaccine in the prime-booster regimen showed clinical signs of infection (Fig. 3B). On the other hand, although 70% and 60% of animals submitted to a single type immunization, with one dose of the pE1D2 or YF17D-D2 vaccines, respectively, survived the DENV2 challenge (Fig. 3A), 50% and 80% of these mice presented morbidity, respectively (Fig. 3B). Furthermore, although most mice immunized with the prime-booster protocol using the pcTPA control plasmid [pcTPA (2d)+YF17D-D2 (1d)] also survived the virus challenge (80%) (Fig. 3B), the majority of them showed remarkable clinical signs of infection (Fig. 3B).

**Figure 4:**
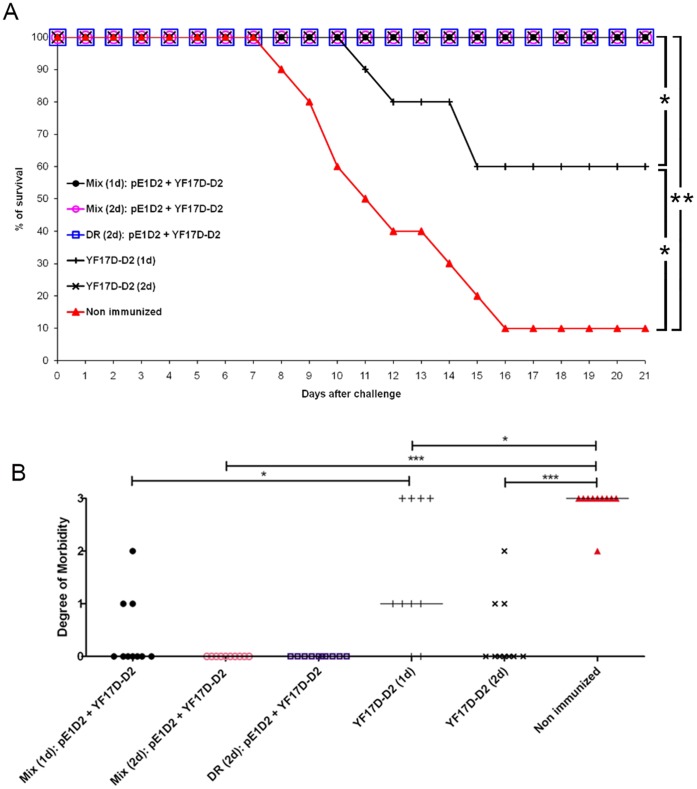
The protective efficacy of vaccination with pE1D2 and YF17D-D2 against DENV2 inoculated simultaneously. Groups of Balb/c mice (n = 10) were immunized by the i.m. route with a mixture of these two vaccines (Mix) or by the i.m. inoculation of the pE1D2 plasmid and s.c. administration of the YF17D-D2 chimeric virus simultaneously (different routes, DR). Animals were challenged (i.c.) with the NGC DENV2 and monitored for 21 days after challenge with the record of survival rates (A) and degree of morbidity (B). Asterisks indicate statistically significant differences using the chi-square test in (A) (*p<0.001; **p<0.0001) and the Mann-Whitney test in (B) (*p<0.05; **p<0.01, ***p<0.001). One dose = 1d; two doses = 2d.

Protocols of combined immunizations using simultaneous inoculations also induced 100% survival, regardless of the number of doses (Fig. 4A). Additionally, animals immunized with two doses of these combined vaccines (Mix 2d), showed complete protection against DENV2, without any morbidity (Fig. 4B). On the other hand, 30% of mice immunized with two doses of the YF17D-D2 presented clinical signs of infection (Fig. B). The protective efficacy was also evaluated in animals inoculated simultaneously with the two vaccines administered by different routes (DR) and results revealed the same level of protection (100% survival, without any clinical signs of infection) (Fig. 4).

## Discussion

In the present report, we investigated the immune response and protection induced with a combination of two different vaccine strategies against dengue in mice, using a DNA plasmid (pE1D2) and a yellow fever/dengue chimeric virus (YF17D-D2). Both vaccines contain sequences from the DENV2 envelope gene. The pE1D2 DNA vaccine encodes 80% of the DENV2 envelope protein sequence, corresponding to its ectodomain (domains I, II and III) without the C-terminal stem-anchor region, fused to the t-PA signal peptide [20]. Expression of the recombinant E protein was previously demonstrated *in vitro*, in BHK cells transfected with the pE1D2 plasmid, and results revealed that the t-PA signal sequence was able to mediate secretion of the ectodomain with expected molecular weight [20], which is an important characteristic for efficient humoral immune response activation by DNA vaccines [26]–[28]. The YF17D-D2, in its turn, is a live attenuated chimeric virus derived from the yellow fever 17D vaccine virus in which the pre-M and E genes were replaced by the corresponding sequences from DENV-2 [21], [29].

The envelope glycoprotein is present on the surface of virus particle and it contains several epitopes that elicit neutralizing antibodies against dengue [7], [30]–[32]. Such antibodies have an important role in dengue immunity, by blocking the binding of virions to cell receptors which culminates in the failure of virus infection and replication, and they have generally been used as a marker for vaccine effectiveness [16]. Most of dengue neutralizing antibodies is directed to the E protein and some studies suggested that the domain III is the major target for the induction of such antibodies [7], [33], since it is the binding domain for the attachment of DENV to cell receptors [6], [31]. However, we previously observed that a DNA vaccine based only on the domain III elicited a weak neutralizing antibody response in mice when compared to full-length ectodomain-based vaccine [20]. Besides, other reports have also suggested that domains I and II contain epitopes which seem to be important for the generation of neutralizing antibody against DENV [30], [34].

We observed that immunization with one or two doses of the pE1D2 DNA vaccine induces detectable levels of neutralizing antibodies against DENV2, ranging from 1∶8 to 1∶240. In a previous work with this DNA vaccine we observed a significant increase of neutralizing antibody levels after challenge with DENV2 by the intracerebral route, indicating the activation of immunological memory with rapid and strong secondary hummoral response, which may also contribute for protection [20]. Mice immunized with the YF17D-D2 chimeric virus also presented neutralizing antibodies, mainly those animals inoculated with two virus doses, (PRNT median = 349). However, such humoral immune response increased remarkably when animals were inoculated with the combination of the two vaccine strategies, most of them reaching the maximal antibody level detected in our PRNT assay (titers ≥640). The synergistic effect on the immune response was observed even when experiments were performed with the prime/boost immunization protocol using only one dose of each vaccine. However, the highest antibody levels were detected in animals receiving two doses of the DNA plasmid followed by one dose of the chimeric virus. Moreover, the immunization with the reversed prime/boost regimen (chimeric virus/DNA vaccine) was also effective and induced an even more efficient neutralizing antibody response. Some reports also demonstrated that DNA vaccines combined with other immunization strategies induces higher humoral immune responses against different pathogens, including dengue viruses, when compared to each approach tested singly [17], [18], [35]–[40]. The prime/boost approach using DNA vaccine has been investigated with different combinations, including subunit vaccines, inactivated pathogens as well as the use of live viral vectors [17], [36], [38]–[41]. However, as far as we know, our work is the first report of using the combination of a DNA vaccine and a chimeric yellow fever/dengue virus for induction of an immune response against DENV. Besides, regarding dengue vaccines, none of the trial products tested in humans showed yet to be completely protective, thus indicating the need to pursue new strategies to reach a broader protection.

In addition, the simultaneous inoculation of the pE1D2 DNA vaccine with the YF17D-D2 chimeric virus also revealed a potent synergistic effect on the neutralizing antibody response in mice, regardless of the immunization route, with an unique formulation of both vaccines delivered by the i.m. route (Mix) or with inoculations by the i.m. and s.c. routes for the DNA vaccine and the chimeric virus, respectively (DR). Previous reports using DNA vaccine and other strategies, such as recombinant proteins or inactivated virus, also showed an increase in antibody response when both vaccines were administered simultaneously [37], [38]. Apparently, the use of two different immunization strategies, either by a prime/boost regimen or with simultaneous administrations, lead the antigen to the mouse immune system in a distinct way, which may deliver it to different antigen presenting cells, thus, eliciting different immune responses either in its magnitude and/or its quality. This can be the reason why chimeric virus YF17D-D2 inoculations did not induce strong IFN-γ responses as we detected in animals immunized with the DNA vaccine pE1D2.

Although most of the studies concerning the immune response induced towards the E protein are based on antibody production, other reports suggests that the activation of T cells with of IFN-γ expression may also contribute to protection against dengue [42]–[45]. In our work, we observed a robust production of IFN-γ by splenocytes collected from mice immunized with the pE1D2 DNA vaccine and stimulated with the peptide E_331–339_, which was previously described as an immunodominant cytotoxic CD8^+^ T cell epitope in Balb/c mice specific for the serotype 2 [23]. Similar results were detected with cells obtained from animals immunized with the combination of the two vaccines (pE1D2 and YF17D-D2), either in the prime/boost system or with simultaneous inoculations. On the other hand, mice vaccinated only with the YF17D-D2 chimeric virus showed a significantly lower IFN-γ response. The induction of robust cellular immune responses in animals inoculated with DNA vaccines against different pathogens has been widely described [46], [47]. However, different from what we observed in the antibody production, we did not detected a synergistic effect in the IFN-γ-positive T cell response in mice immunized with the combination of the two vaccines. In fact, the number of SFC in the ELISPOT assay with splenocytes collected from these mice were similar to what we detected in animals immunized with only the pE1D2, suggesting that such response was induced predominantly by the DNA vaccine. The cellular immune response is a key aspect for the induction of a long-lasting immunologic memory [48]–[50]. Therefore, these results reinforce the idea that the combination of the two immunization strategies can lead to a more efficient vaccine. This is an important finding, since recently results were published concerning the protective efficacy of the tetravalent Sanofi-Pasteur vaccine, also based on chimeric yellow fever/dengue virus, in a phase 2 trial with children [51]. Authors showed that, although children presented high levels of dengue-2 neutralizing antibodies, they were not protected against this virus. Such result reveals that other branch of the immune response should be investigated and that the induction of a robust cellular response with production of IFN-γ may be important for protection.

In accordance to the immune response detected in vaccinated mice and discussed above, the challenge experiments with a lethal dose of DENV2 also revealed in the present work that the combination of the two different immunization strategies were more protective than each vaccine administered singly, specially when two doses of the DNA vaccine pE1D2 were administered. These animals were fully protected, without presenting any clinical sign of the dengue infection, either in mouse groups submitted to prime/boost immunizations [pE1D2(2d)+YF17D-D2(1d) or YF17D-D2(1d)+pE1D2(2d)] or with simultaneous inoculations [Mix (2d): pE1D2+ YF17D-D2 or DR: pE1D2+ YF17D-D2].

Although symptoms manifested in mice inoculated with dengue virus by the intracerebral route are not exactly the same as observed in humans, it has been reported that infection with dengue can also lead to encephalitis in some fatal cases, with detection of virus antigens or the virus RNA in the brain [52]-[54]. Therefore, such evidences indicate the involvement of the central nervous system in the pathogenesis of dengue. Moreover, we choose this challenge model because it is the most widely used model for vaccines tests against dengue virus [12], [20]–[21], [24], [27], [55]–[56]. Hence, results presented in the present work can be compared to those described in several studies. Most animals were challenged 15 days after the last immunization because in older animals the effect of the dengue inoculation is reduced. However, another mouse group simultaneous inoculated with the two vaccine strategies was also challenged 30 days after the last immunization and all vaccinated mice survived virus infection, without the appearance of any clinical sign of infection (data not shown).

The YF17D-D2 chimeric virus used in our experiments was constructed in Brazil [21] by the exchange of the prM-E genes. The Sanofi-Pasteur also developed a dengue vaccine based on a tetravalent formulation of prM-E chimeric viruses and this vaccine has undergone extensive clinical testing [14], [15], [51], [57]. Such trials revealed the necessity of three doses with several month intervals to achieve a balanced antibody response against all four dengue serotypes [14], [15]. The administration of several doses for long periods may represent one problem for vaccination in dengue endemic regions, since it can leave the population more susceptible to the development of severe disease until it became immunologically covered and protected. Therefore, combined immunization of a chimeric yellow fever/dengue virus with a DNA vaccine, as proposed in the present work using DENV2 as a proof of principle, may circumvent such difficulty for induction of robust immune responses against all serotypes, reducing the number of doses and/or the vaccination intervals. Moreover, it is important to note that when the pE1D2 or the YF17D-D2 vaccines were tested alone [20]–[21] they do not induced fully protection against dengue, which was only achieved after immunization of both strategies in the same animal. Furthermore, the combination of the two different immunization strategies administered simultaneously (Mix or DR groups), instead of the prime/boost regimen, is an attractive approach for vaccination campaigns, since it can facilitate the logistic for inoculations using a single formulation. On the other hand, the reversed prime-boost system also opens a perspective for the use of a DNA vaccine as a booster, particularly in individuals who do not achieve satisfactory neutralizing antibody levels against the four serotypes after immunization with the yellow fever/dengue chimeric virus. In this case, the booster DNA vaccine may be administered as monovalent or tetravalent formulations. However, further studies will be necessary for testing such hypothesis using tetravalent DNA vaccine and chimeric virus formulations.

Another problem concerning dengue tetravalent vaccines based on live attenuated viruses is the possibility of viral interference, leading to heterogeneous immune responses against each serotype [9]. On the other hand, DNA vaccine appears not to cause interference upon combined immunization and in fact it seems to be more effective when combined to other vaccine strategies [17]–[19]. Although virus interference may still occur when the two strategies are used, the DNA immunization may also help for balancing the immune response induced against each serotype, providing a booster response for one or some virus when necessary. However, further experiments will be conducted to test if the interference phenomenon may be overcome in a tetravalent vaccine consisting of chimeric virus YF17D-dengue and DNA vaccines.

Additionally, the Mix regimen used in the present work represents an interesting strategy because it can reduce the number of doses. We used the term “dose” as the number of immunization in each animal at a specific time point. Although mice received the double concentration of each vaccine in the Mix (2d), for example, they were given simultaneously, thus maintaining interval between doses (two weeks). Furthermore, in the case of Mix (1d), although three mice presented clinical sings of infection, this system was more protective when we compared to pE1D2 (1d) or YF17D-D2 (1d). Moreover, when we think about the immunization schedule, the Mix (1d) can be considered also more protective than the YF17D-D2 (2d), since it attained the same result (100% survival with 30% morbidity) as the second group but in a single vaccination time.
